# Detecting and mitigating simultaneous waves of COVID-19 infections

**DOI:** 10.1038/s41598-022-20224-5

**Published:** 2022-10-06

**Authors:** Sebastian Souyris, Shuai Hao, Subhonmesh Bose, Albert Charles III England, Anton Ivanov, Ujjal Kumar Mukherjee, Sridhar Seshadri

**Affiliations:** 1 Lally School of Management, Rensselaer Polytechnic Institute, Troy, NY 12180 USA; 2 Department of Business Administration, Gies College of Business, University of Illinois Urbana-Champaign, Urbana, IL 61820 USA; 3 Department of Electrical and Computer Engineering, Grainger College of Engineering, University of Illinois Urbana-Champaign, Urbana, IL 61801 USA; 4 OSF HealthCare Heart of Mary Medical Center, Urbana, IL 61801 USA; 5 Carle Illinois College of Medicine, University of Illinois Urbana-Champaign, Urbana, IL 61820 USA

**Keywords:** Health policy, Viral infection, Statistical methods, Risk factors

## Abstract

The sudden spread of COVID-19 infections in a region can catch its healthcare system by surprise. Can one anticipate such a spread and allow healthcare administrators to prepare for a surge *a priori*? We posit that the answer lies in distinguishing between two types of waves in epidemic dynamics. The first kind resembles a spatio-temporal *diffusion* pattern. Its gradual spread allows administrators to marshal resources to combat the epidemic. The second kind is caused by super-spreader events, which provide *shocks* to the disease propagation dynamics. Such shocks simultaneously affect a large geographical region and leave little time for the healthcare system to respond. We use time-series analysis and epidemiological model estimation to detect and react to such simultaneous waves using COVID-19 data from the time when the B.1.617.2 (Delta) variant of the SARS-CoV-2 virus dominated the spread. We first analyze India’s second wave from April to May 2021 that overwhelmed the Indian healthcare system. Then, we analyze data of COVID-19 infections in the United States (US) and countries with a high and low Indian diaspora. We identify the Kumbh Mela festival as the likely super-spreader event, the exogenous shock, behind India’s second wave. We show that a multi-area compartmental epidemiological model does not fit such shock-induced disease dynamics well, in contrast to its performance with diffusion-type spread. The insufficient fit to infection data can be detected in the early stages of a shock-wave propagation and can be used as an early warning sign, providing valuable time for a planned healthcare response. Our analysis of COVID-19 infections in the US reveals that simultaneous waves due to super-spreader events in one country (India) can lead to simultaneous waves in other places. The US wave in the summer of 2021 does not fit a diffusion pattern either. We postulate that international travels from India may have caused this wave. To support that hypothesis, we demonstrate that countries with a high Indian diaspora exhibit infection growth soon after India’s second wave, compared to countries with a low Indian diaspora. Based on our data analysis, we provide concrete policy recommendations at various stages of a simultaneous wave, including how to avoid it, how to detect it quickly after a potential super-spreader event occurs, and how to proactively contain its spread.

## Introduction

As of August 10, 2022, COVID-19 has claimed more than 6.4 million lives worldwide (https://covid19.who.int/). The spread and the impact of the disease since early 2020 have not been uniform over time. Data on infection spread across the globe exhibit wave-like patterns; the cases often grow rapidly for a period of time before mitigating measures arrest the growth and reverse that trend. A closer look at these waves reveals that not all waves have similar spatio-temporal signatures. In some, the temporal pattern of the waves among the various states within a country rise, but not simultaneously. As will be evident from our plots, India’s first wave of COVID-19 infections in 2020 fits this pattern, where infection progressively propagated from one state to another. As a result, the peaks of the waves in different parts of a nation occurred at different times. The demand for healthcare resources during such a *diffusion*-style infection spread gets distributed over time. A carefully crafted policy can enable resource-sharing to manage healthcare needs. The picture is very different in waves that rise *simultaneously* in every state within a nation. A case in point is India’s second wave in the summer of 2021. Fueled by the highly transmissible B.1.617.2 Delta variant of the SARS-CoV-2 virus, the infection load grew almost simultaneously across multiple states in India^1,2^. The concurrency of the growth across the states overwhelmed the national healthcare system. Shortage of hospital beds, staff, ventilators, etc., became a reality everywhere throughout the whole nation. The Delta variant was identified as a variant of concern by the World Health Organization on May 11, 2021. It is 1.64 times more transmissible than the B.1.1.7 (Alpha) variant, or 2.3–3.2 times more transmissible than the wild-type SARS-CoV-2 virus. Hospitalization rates have been 1.5–1.8 times higher than that after infection with the Alpha variant and 2.3–2.8 times higher after infection with the wild-type virus^3^.

The infection pattern in the United States (US) tells a similar story. As our plots will reveal, the first and the second waves in the US in 2020 and early 2021 had diffusion-type growth patterns. On the other hand, the third wave later in the summer of 2021 had a simultaneous uptick across all states. This growth in the US led to significant policy shifts regarding mask mandates, travel restrictions, etc. The effect was less dire than that in India. The Delta variant reached America’s shores after peaking in India, and the US had already vaccinated a significant portion of its adult population by then. The healthcare system was decidedly better prepared. Nevertheless, it faced difficulties in various states, especially those where the vaccination rates were low at that point.

### Our contributions

Given the very different natures of the two types of waves in epidemic spreads, in this paper, we ask three questions. First, how do these simultaneous waves originate? Second, can one identify signatures of these waves early enough to issue warning signs to proactively plan for them? Third, what measures can one take to avoid such waves and what policies one can adopt when the peaks from simultaneous waves are imminent? We systematically answer these questions using statistical data analysis with COVID-19 data from India, US and other countries. Concretely, the contributions of this paper are three-fold.

- We identify two important origin stories for simultaneous waves. First, they emerge due to super-spreader events, where large crowds congregate from multiple regions in a shared location and subsequently return to their respective locations. The infection spreads among the attendees; then, almost simultaneously, it reaches those diverse geographical regions when these attendees return home. We provide evidence that the Kumbh Mela festival in India in 2021 was such a super-spreader event on a national scale. Second, significant international travel from a country that is experiencing a surge in cases from a highly contagious variant can also lead to simultaneous waves in another nation. Indeed, our data analysis reveals that the Delta variant-induced uptick in COVID-19 cases in the US can be linked to international travel from India, bringing the infection to multiple states within the country, simultaneously.
- We propose two methods to detect early signs of simultaneous waves. The first among these utilizes an estimation procedure with a susceptible-infected-removed (SIR) multi-area epidemiological model. As we will show, this model estimation procedure fits the data of infection growth well when the waves are not simultaneous across the nation. Trying the same in the early days with simultaneous waves results in a poor fit to data. This lack of fit can be viewed as a statistical control tool: a significant prediction error of the number of new cases is a signature of simultaneous waves. Second, we show that even a simple count that tracks the number of new cases per location during the early days of the simultaneous growth is a good indicator of simultaneous waves.
- Using our analysis of COVID-19 data from India, the US, and other countries, we propose four concrete policy recommendations to counter simultaneous waves. Namely, they are: (1) identify variants of concerns through genomic testing of new cases, possibly through international coalitions, that may be on the rise in any part of the world^2,4,5^; (2) limit and manage potential super-spreaders events; (3) detect an imminent simultaneous rise in cases across a nation early enough using one of our simple tests to prepare for it, e.g., by restricting internal mobility, enforcing masks usage, allocating healthcare resources, etc.; (4) monitor and possibly restrict travel from countries with rising infections levels and implement stringent testing protocols at airports to limit the influx of new infections.

The Delta variant has been overtaken by the Omicron variant, a more transmissible but less virulent mutation of the SARS-CoV-2 virus^6^. It is also quite possible that COVID-19 will not be the last pandemic in this increasingly connected world. Hence, our study of the origins of simultaneous waves provides valuable insights into the mechanics of epidemics that do not exhibit classic diffusion-type growth patterns. Our recommendations are actionable; they should allow policy-makers to counter such waves effectively during various stages of a simultaneous wave. However, our recommendations are based on publicly available data that was not collected via systematic experimental design. As a result, our conclusions are only as good as the quality of the data itself. In addition, our conclusions are purely associative in nature. We do not claim causal relationships between the plausible wave origins and the wave patterns. Establishing such a relationship requires a more sophisticated analysis, controlling for a range of confounding covariates, which we relegate to future endeavors.

**Figure 1:**
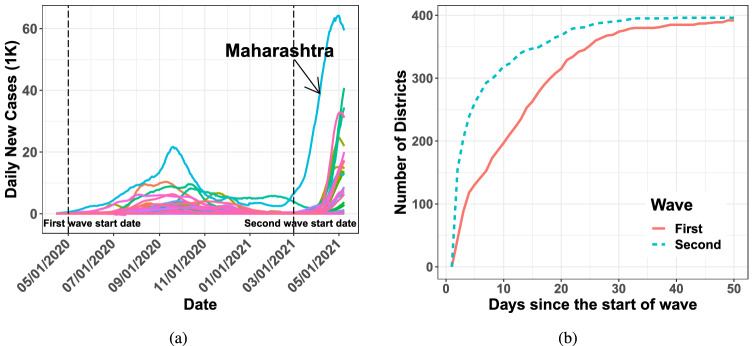
Plot (**a**) shows the number of daily new COVID-19 cases for all the states of India, smoothed via 14 days rolling average. Plot (**b**) illustrates the number of districts with more than five cumulative new COVID-19 cases since the start dates of the two waves—first wave since 05/01/2020 and second wave since 03/01/2021.

## The super-spreader behind India’s second wave in 2021

COVID-19 in India was on the decline between December 2020 and February 2021. A rapid spike in infections appeared in late March 2021. The swiftness of the onslaught left millions suffering from significant shortages in emergency medical supplies and hospital capacities. The death toll from COVID-19 in India on May 25, 2021, became the highest for any nation in a single day. This surge in cases is the second wave of COVID-19–the first wave in mid-2020 had largely dissipated by then. The second wave differs from the first wave in two ways. First, the increase in infections during the second wave coincides with the growth of the Delta variant that was first identified in the state of Maharashtra in late 2020. Second, the growth of new infections in the second wave is sharp and almost simultaneous over most parts of India. The first wave, on the other hand, resembles a spatio-temporal diffusion. Consider the daily number of COVID-19 infections in various states in Fig. 1a, drawn using district-wise COVID-19 positivity results from https://www.covid19india.org/. We sub-select 394 districts among the 675 districts in India with complete data of daily new positive cases from 05/01/2020 to 05/08/2021; see our data repository. Then, we aggregate the data from the districts to compute state-wise infection levels in Fig. 1a. Notice that the state of Maharashtra shows a steady growth of infections since January 2021, where the Delta variant was first detected in late 2020. In all other states, the second wave does not appear till April 2021, when the infections abruptly rise everywhere. We consider 05/01/2020 to 10/31/2020 and 03/01/2021 to 05/08/2021 as the date ranges for the first and the second waves, respectively. The waves’ start dates are approximated. There is no official institution that states the start date of a wave, as, for example, the World Health Organization declared the COVID-19 outbreak to be a pandemic on 03/11/2020. Different sources cite slightly different dates around the waves’ start dates that we name. With these date ranges, the number of districts with $\ge 5$ cumulative number of COVID-19 infections since the starts of the two waves in Fig. 1b also underscores the concurrence in infection growth across the country.

**Figure 2:**
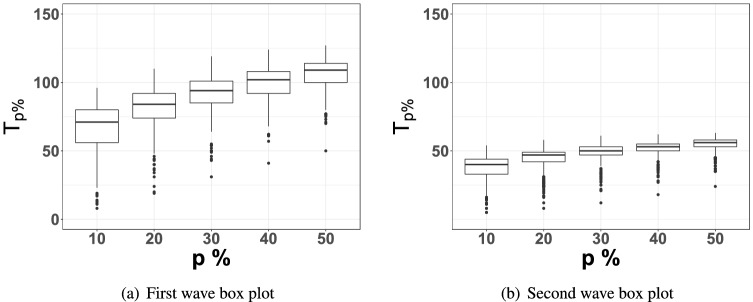
Box plot in (**a**) portrays $T_{p\%}$ across 394 Indian districts during the first wave (starting in 05/01/2020). The box plot in (**b**) portrays the same for the second wave (starting in 03/01/2021).

To further illustrate the simultaneity of the second wave, consider the time elapsed $T_{p\%}$ since the start dates of the two waves to reach $p\%$ of their levels on 09/15/2020 and 05/08/2021, respectively. The box plot of $T_{p\%}$ for $p=10,20,30,40,50$ in Fig. 2 clearly show a larger spread among $T_{p\%}$’s in the first wave (Fig. 2a) compared to that in the second wave (Fig. 2b).

**Figure 3:**
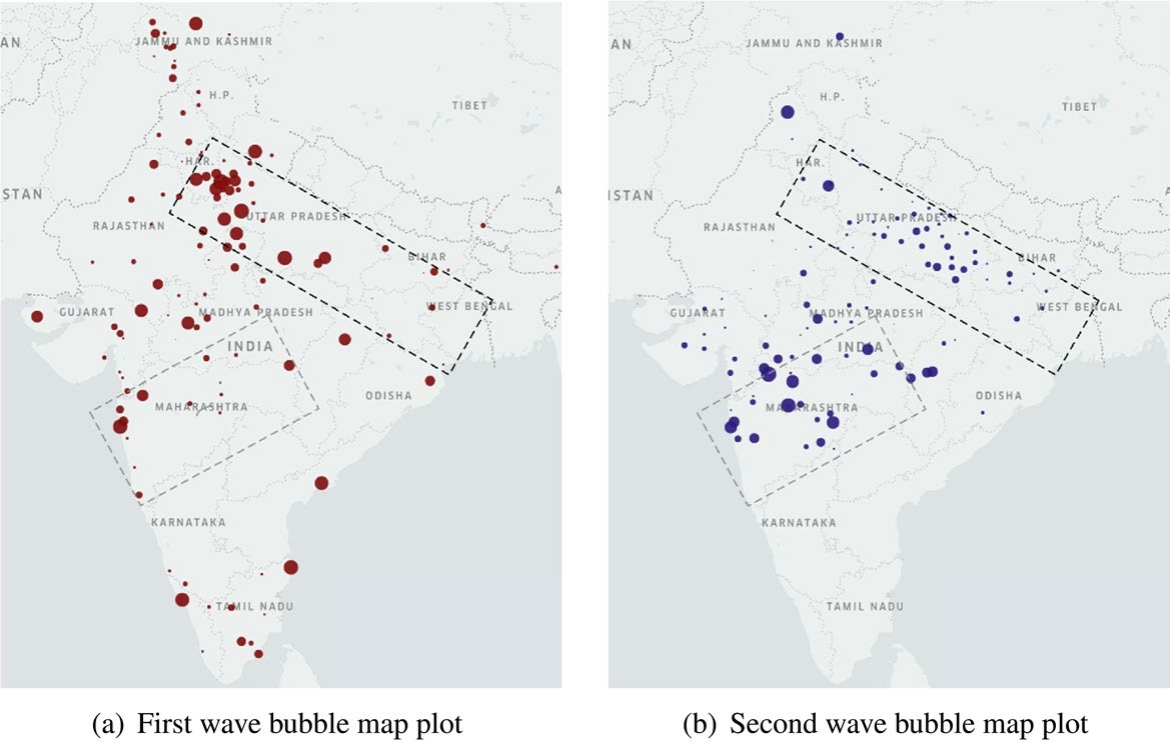
Bubble map plots of $\kappa _\star$ for Indian districts that have $\kappa _\star > 0$ for the first wave in (**a**) and the second wave in (**b**). The size of the bubble indicates the magnitude of $\kappa _\star$. The dotted areas identify Maharashtra and the Ganges belt.

### Origins of the COVID-19 waves in India via time-series analysis

Several tools can be used to conduct forensic analysis of the patterns of spread. Here, we leverage cross-correlation analysis of time-series data for the same. Specifically, cross-covariance of time-series data can shed light on the causal effect of one time-series on another. We use cross-covariance among the time series of COVID-19 infections across 394 districts to identify this causal effect, ignoring the impacts of possible inadequate testing and erroneous reporting.

Consider the sequence of new daily COVID-19 infections in district *i* on day *t* as $\rho ^i_t$, where *t* is measured as days since the start dates of each wave. Then, the cross-covariance function over *T* time-periods is described by

1 $$\begin{aligned} \mathscr {C}(i,j; \kappa ) := \frac{1}{T}\sum _{t=1}^T \left( \rho ^i_t - \langle \rho ^i\rangle \right) \left( \rho ^j_{t+\kappa } - \langle \rho ^j\rangle \right) , \end{aligned}$$

where $\kappa$ denotes the time-shift of one time-series with respect to the other in calculating the covariance. Here, $\langle \rho ^i\rangle$ and $\langle \rho ^j\rangle$ compute the empirical means of the *T*-length time series $\rho ^i$ and $\rho ^j$, respectively. The value of $\kappa _\star (i, j)$ at which the covariance is maximized between the daily new infections in district *i* and that in district *j* denotes the number of days by which the infection pattern in district *j* roughly lags the pattern in district *i*. We vary $\kappa$ in $[-30, 30]$ with $T=92$ days for the emergence of the first wave and $T=69$ days for the second wave to compute $\kappa _\star (i, j)$. A time-lag between the patterns of district *i* and district *j* does not imply that infected people from district *i* came in direct contact with people in district *j* to drive the spread of COVID-19. However, consistent positive values of $\kappa _\star (i, j)$’s for multiple *j*’s suggests that district *i* is an epicenter of the infection spread.

**Figure 4:**
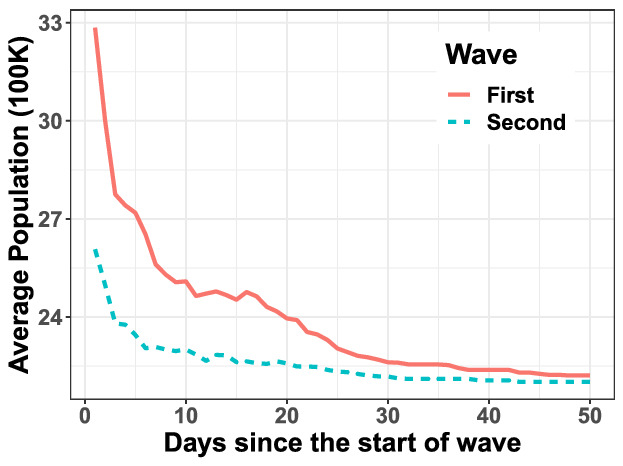
Variation of average population of Indian districts with cumulative number of $\ge 5$ infections since the start of the waves as in Fig. 1b. Start dates of the two waves are 05/01/2020 and 03/01/2021.

Figure 3 portrays a bubble plot of $\sum _{j=1}^N \kappa _\star (i,j)$ over those locations *i* for which this sum is positive. A larger bubble indicates higher likelihood of a location being a source of the infection spread. Figure 3 reveals important differences between the likely epicenters of the two waves. The first wave developed around large cities (possibly from contact with infected people engaged in foreign travel). The second wave largely originates from Maharashtra and, to a smaller extent, the Ganges belt. Figure 4 affirms that the infection spread from higher to lower populated districts during the first wave. Populations of the districts affected through time is much “flatter” during the second wave, suggesting the role of super-spreader events in affecting the nation almost uniformly. Maharashtra as a likely epicenter for the second wave, corroborates our theory about the circulation of the Delta variant, aligned with the findings from genomic sequencing of COVID-positive patients. The remaining epicenters in the Ganges belt point towards the Kumbh Mela festival as the super-spreader event^7–9^. Several cases of COVID-19 infection during the second wave could be contact traced back to the Kumbh Mela gathering^10^.

### Multi-area SIR model for India’s infection dynamics

This section proposes a more proactive way to detect super-spreaders, using a susceptible-infected-removed (SIR) multi-area compartmental model. We provide additional evidence for our hypothesis that super-spreader events played a dominant role in India’s second wave. To do so, we consider an epidemiological diffusion model, estimate its parameters from the district-wise test results, and demonstrate lack of fit during the second wave. To this end, consider the SIR compartmental model, described by

2 $$\begin{aligned} \begin{aligned} S^i(t+1)&= S^i(t) - \beta ^{i}_{\text {int}}(t) I^i(t) S^i(t) - \beta ^{i}_{\text {ext}}(t) I^i_{ext}(t) S^i(t), \\ I^i(t+1)&= I^i(t) + \beta ^{i}_{\text {int}}(t) I^i(t) S^i(t) + \beta ^{i}_{\text {ext}}(t) I^i_{ext}(t) S^i(t) - \gamma I^i(t), \\ R^i(t+1)&= R^i(t) + \gamma I^i(t). \end{aligned} \end{aligned}$$

Here, $S^i(t)$ denotes the fraction of the population in district *i* that is susceptible to the infection on day *t*. Similarly, $I^i(t)$ and $R^i(t)$ are the fractions of the infected population and the removed (recovered or deceased) population in district *i* on day *t*, respectively. Again, we consider $N=394$ districts. Per^11^, we choose $\gamma = \left( 14 \ \text {days}\right) ^{-1}$ as the combined constant rate of recovery and death from COVID-19. Parameter $\beta ^{i}_{\text {int}}$ captures the rate at which the infected population within district *i* contributes to new infections within that district on day *t*. A key innovation in our approach is to *lump* external sources into one, because, the estimation becomes very hard and noisy otherwise. In our model, $\beta ^{i}_{\text {ext}}$ lumps the impact of infections outside of district *i* towards new infections in district *i* on day *t*. The population-weighted external infections seen from district *i* is computed as $I_{\text {ext}}^i(t) := \frac{ \sum _{j:(j,i) \in \mathscr {G}} I^j(t) P^j}{\sum _{j:(j,i) \in \mathscr {G}} {P^j}}$, where $P^j$ is the population of district *j*, per the 2011 Census.

We allow neighbors of district *i* according to a graph $\mathscr {G}$ on *N* nodes as the only places whose infection levels directly impact district *i*’s infections in one day. We construct $\mathscr {G}$ to encode the intuition that geographically distant districts do not directly contribute to each other’s infection spread within one day, unless through long-distance travels such as those on airplanes. To construct $\mathscr {G}$, we first add an edge between districts *i* and *j*, if the distance between the centroids of these districts $d^{i,j}$ is below 1594.5 km. We compute $d^{i,j}$ using the great circle distance formula^12^ applied to the latitudes and longitudes of the centroids from the Kaggle data hub^13^. Then, we add edges between any two districts that contain airports and have at least one direct flight between them. For the flight network, we use the origin-destination airport pairs from^14^. The resultant graph $\mathscr {G}$ then has 4599 edges, much less than that in a fully-connected graph over 394 nodes.

We use two sets of data–the cumulative fraction of COVID-19 cases $Q^i$ on $t=1$ in district *i* and the fraction of new COVID-positive cases $\Delta ^i(t)$ in district *i* on days $t=1, \ldots , T$. Specifically, *Q*’s yield

3 $$\begin{aligned} S^i(1) = 1 - Q^i, \ I^i(1) = (1-\gamma ) Q^i, \ R^i(1) = \gamma Q^i, \end{aligned}$$

that are then propagated using $\Delta$’s via

4 $$\begin{aligned} \begin{aligned} S^i(t+1)&= S^i(t) - \Delta ^i(t),\\ I^i(t+1)&= I^i(t) + \Delta ^i(t) - \gamma I^i(t),\\ R^i(t+1)&= R^i(t) + \gamma I^i(t). \end{aligned} \end{aligned}$$

**Figure 5:**
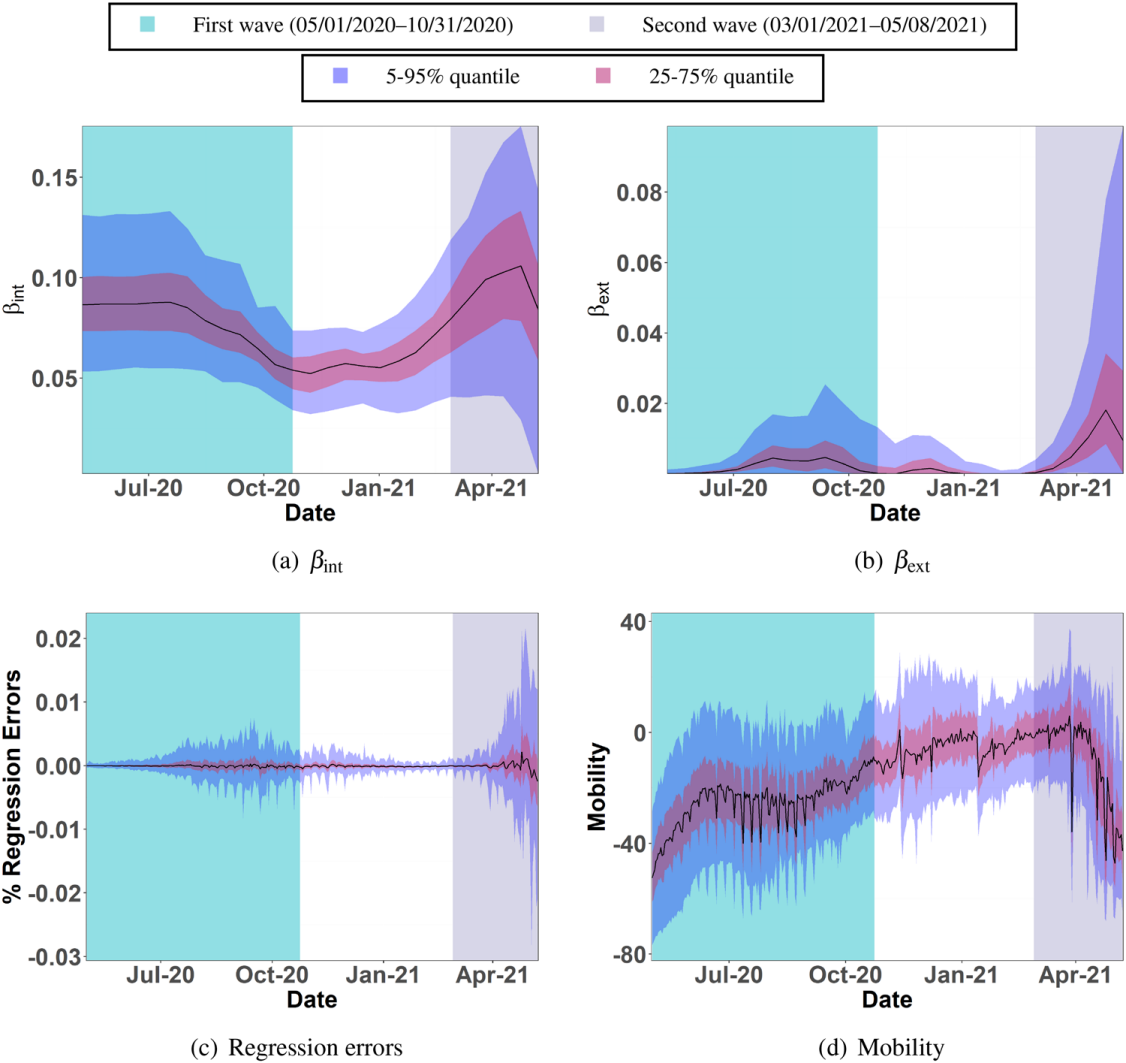
Plots (**a**) and (**b**) capture the quantiles of $\beta _\text {int}$’s and $\beta _\text {ext}$’s across Indian districts over 2-week time windows from 05/01/2020 to 05/08/2021. Plot (**c**) displays the regression errors in explaining the emergence of daily new infections with the estimated $\beta$’s. Plot (**d**) shows the quantiles of mobility variations across districts.

We now describe our procedure to estimate the $\beta$ parameters from the time-series of $S^i,I^i,R^i$ over 05/01/2020–05/08/2021. Since we expect mobilities, and in turn the $\beta$’s, to change gradually, we assume that $\beta ^{i}_{\text {int}}, \beta ^{i}_{\text {ext}}$ remain constant over 2-week periods. Denote them as ${\beta }[1], \ldots , {\beta }[26]$, where ${\beta }[\tau ]$ stands for ${\beta }_\text {int}(t), \beta _\text {ext}(t)$ across all districts from $t = 14\tau -13$ to $t = 14\tau - 1$ days. Then, we minimize $\varphi$ to estimate ${\beta }[1], \ldots , {\beta }[26]$, where

5 $$\begin{aligned}&\varphi \left( {\beta }[1], \ldots , {\beta }[26]\right) \\&\quad := \sum _{i=1}^N \sum _{\tau =1}^{26} \sum _{t=14\tau - 13}^{14\tau } \big (\Delta ^i(t) - \beta ^{i}_{\text {int}}[\tau ] I^i(t) S^i(t) - \beta ^{i}_{\text {ext}}[\tau ] I^i_{\text {ext}}(t) S^i(t) \big )^2 \\&\qquad + \lambda \sum _{\tau =1}^{25} \sum _{i=1}^{N} \left[ \left( {\beta }^i_\text {int}[\tau +1]) - {\beta }^i_\text {int}[\tau ]) \right) ^2 + \left( {\beta }^i_\text {ext}[\tau +1]) - {\beta }^i_\text {ext}[\tau ]) \right) ^2 \right] + \rho \sum _{\tau =1}^{26} \sum _{i=1}^{N} \beta _\text {ext}[\tau ]^2. \end{aligned}$$

The first summand in (5) equals the regression error in explaining the emergence of daily new infections using $\beta$’s. The second and the third summands in (5) penalize deviations of $\beta$’s across consecutive 2-week periods. This penalty encodes the intuition that $\beta$’s should vary gradually over time. The last summand in the definition of (5) adds a regularization that reduces the tendency of the estimator to explain the emergence of new infections solely using external infections. For the estimation process, we use $\lambda = \rho = 10^{-3}$.

The quantile plots of $\beta _\text {int}$’s in Fig. 5a and $\beta _\text {ext}$’s in Fig. 5b across the districts over time reveal the two waves of COVID-19 infections. The jump in $\beta _\text {ext}$ during the second wave appears particularly pronounced, compared to that during the first wave. Despite an explicit penalty on large $\beta _\text {ext}$’s, the estimation favors $\beta _\text {ext}$’s to explain the emergence of new infections, without a similarly sharp increase in $\beta _\text {int}$’s. The quantiles of the regression errors over time in Fig. 5c during the second wave are also much higher than those during the first wave.

One can view regression estimation of the SIR model as an exercise to decompose the disease dynamics into a systematic component and a shock component. The systematic component captures the predictable dynamics of the disease, while the shock captures the impact of *un-modeled* components that the predictors fail to explain. The regression errors and the size of $\beta _\text {ext}$ encode this shock. Evidently, the suddenness and the extent of the shock in India’s second wave is much larger than that in the first wave. Therefore, these errors and $\beta _\text {ext}$ can serve as *indicators* of the presence of a large shock to the normal infection diffusion process. Super-spreaders on a national scale have the potential to substantially alter the infection dynamics sufficiently to generate an aberration in regression estimates. This aberration (seen through $\beta _\text {ext}$ and regression errors) can then be viewed as a signature of a super-spreader event in data.

In Fig. 5d, we plot the quantiles of average mobility across districts over time. To gauge the district-wise mobility of the Indian population, we use data from the Google COVID-19 Community Mobility Reports^15^ that catalogs variations in the population’s activities such as visitations to parks, grocery and pharmacy, retail and recreation, transit stations, and workplaces. Specifically, the data reports the level of these activities compared to the baselines computed as the median values over 01/03/2020–02/06/2020, measured from anonymized cellphone data. We calculate the mobility levels as the average of the above activity levels in the five reported categories for our plots. Note that during the second wave, $\beta _{\text {int}}$ and $\beta _{\text {ext}}$ increase even though mobility decreases. If the model were a good fit during the second wave, one expects $\beta _{\text {int}}$ to correlate positively with mobility because $\beta _{\text {int}}$ captures the effect of the virulence of the virus and the average frequency of contacts that a susceptible person has with the infected population. The estimated $\beta _{\text {int}}$ parameters do not fit that trend with mobility, adding further evidence to the inadequacy of the model to capture the second wave. We remark that one can do a more formal change-point detection analysis; see^16^ for an interesting use of likelihood ratio-based test to detect epidemic waves. Running a similar analysis on our data corroborates the conclusions drawn from $\beta _{\text {int}}$’s. Details of our analysis are available at https://github.com/Ujjal-Mukherjee/India-Second-Wave----Change-Point-/tree/main

In summary, super-spreader-driven infection spread is characterized by the simultaneity of surge across locations (as in the cross correlation-based analysis) and the relatively high shock component of the diffusion model (as in the SIR analysis). Both pieces of evidence support the hypothesis that India’s second wave between April and June 2021 was driven by a super-spreader event. The Kumbh Mela defines the foremost candidate due to its concurrency with the start of the second wave and how well our “origin” locations match with the extent of participation in this event from the various states.

**Figure 6:**
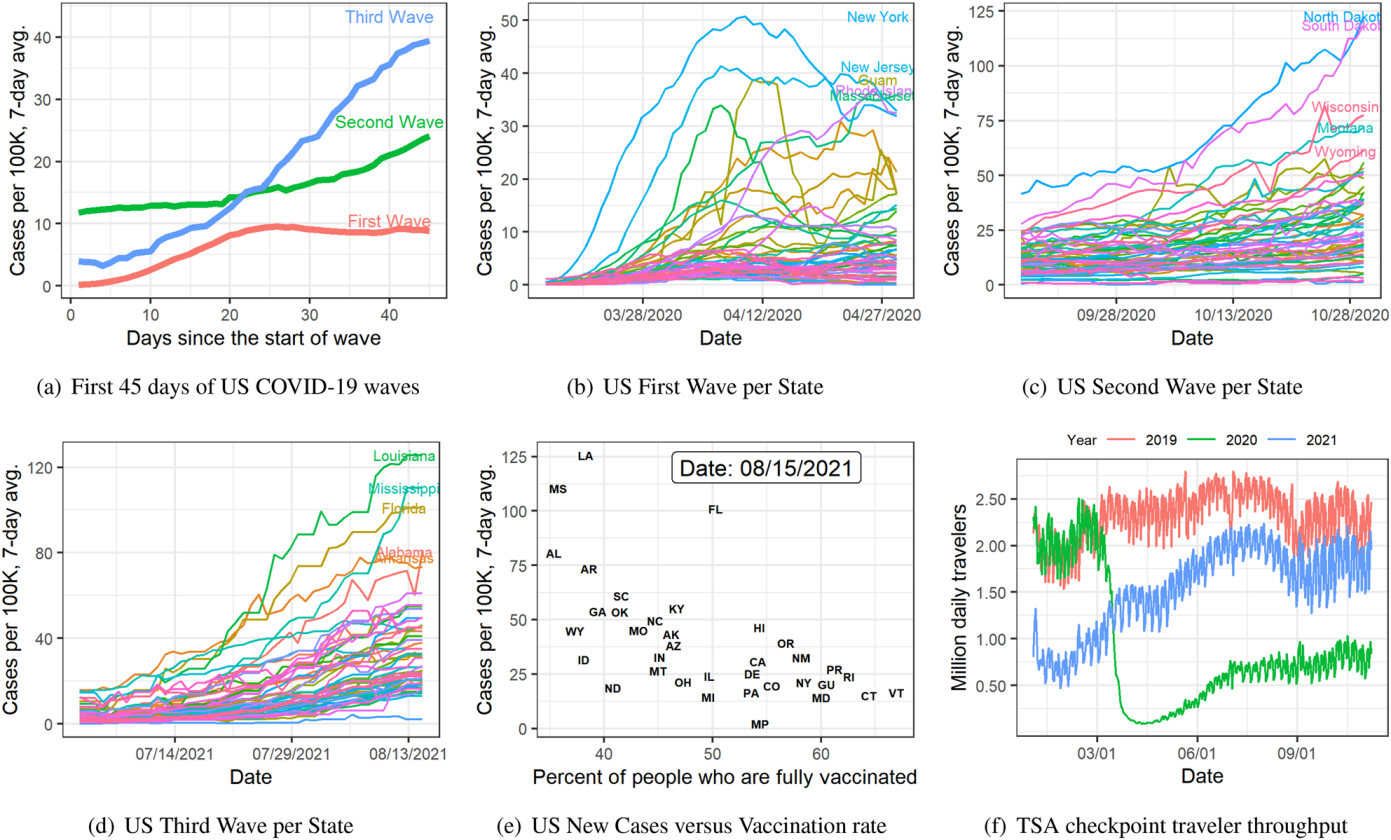
Plot (**a**) shows the seven days rolling average new cases per 100 thousand people during the first 45 days of the US first wave (03/15/2020–4/19/2020), the US second wave (09/15/2020–10/30/2020), and the US third wave (07/01/2021–2021/08/15). Plots (**b**), (**c**), and (**d**) show the seven days rolling average new cases per hundred thousand people during the first 45 days of the first, second, and third waves respectively by state. (**e**) New cases per 100,000 people versus percent of people who are fully vaccinated (have second dose of a two-dose vaccine or one dose of a single-dose vaccine) based on the state where recipient lives on 4th day of the third wave (08/15/2021). Plot (**f**) displays the Transportation Security Administration (TSA) checkpoint travel numbers for years 2019, 2020, and 2021 on the same weekday. Source cases data^17^, vaccines data^18^, TSA traveler throughput data^19^.

## COVID-19 waves in the US: detecting wave propagation of super-spreaders

We now shift gears and focus on the analysis of COVID-19 data from the US. Until 08/15/2021, the US experienced three waves. The key object of our interest is the third wave. Similar to that of India’s second wave, US’s third wave also shows a simultaneous growth in infections across the nation (see Fig. 6d), that does not appear similar to classic diffusion patterns. The infection growth during the first and second waves, on the other hand, are not that simultaneous across the states, as Fig. 6b,c reveal. Moreover, the infection growth during the third wave is faster than in the first two waves, as Fig. 6a uncovers. Here, we compare the seven-days rolling average of new cases per 100,000 people during the first 45 days of the first wave (03/15/2020–04/29/2020), the second wave (09/15/2020–10/30/2020), and the third wave (07/01/2021–2021/08/15). The speed of infection growth can perhaps be attributed to the higher transmissibility of the Delta variant^20^, but the simultaneity of that growth across the states cannot be likewise attributed. Unlike the Indian experience, there was no obvious super-spreader event in the US during this wave. In what follows, we build a hypothesis about the origin of this simultaneous infection growth and provide evidence for that hypothesis with data. Needless to say, the multi-area SIR model will not fit this data. On top of this, we believe that once a super-spreader event happens it transmits like a super-spreader due to travel to other countries. We test that hypothesis in this section.

**Figure 7:**
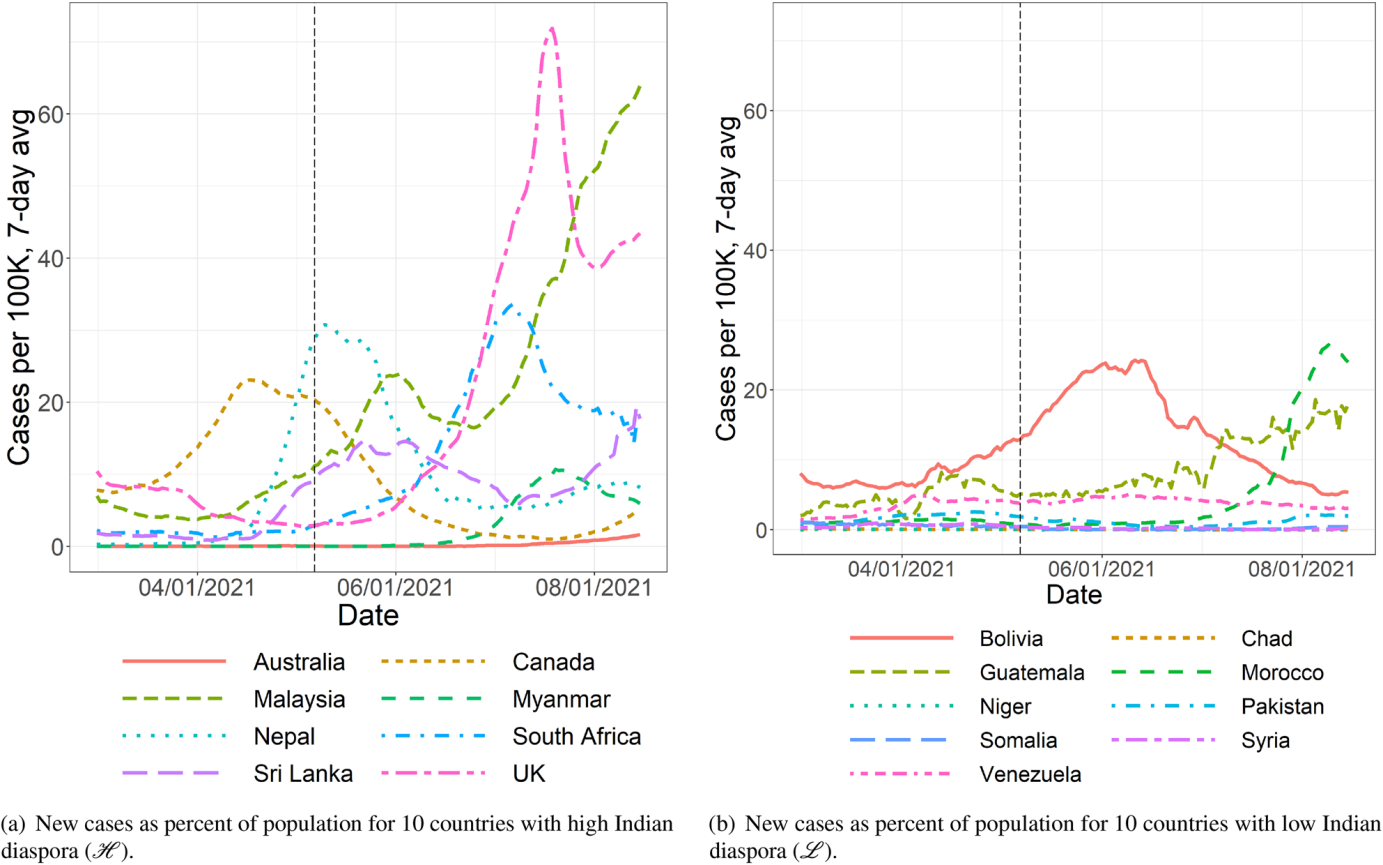
Plot (**a**) shows new cases of COVID-19 infections in 10 countries with high Indian diaspora in $\mathscr {H}$. Plot (**b**) shows selected 10 countries with low Indian diaspora in $\mathscr {L}$.

The third wave of COVID-19 in the US has been driven by the rise of the Delta variant^20^. This variant originated in India and migrated to other countries. As the number of daily infections clocked upwards of 300K with $\sim$4K daily deaths in May 2021 in India, many countries announced India-specific travel restrictions, US being one among these countries. Such announced restrictions often spur quick migrations of people who seek to avoid travel delays from said restrictions. We posit that such travels from India during this period led to the simultaneous rise in Delta variant-based COVID-19 infections in the US. Even if only a minor fraction of the travelers from India on the airplanes to the US were infected, such travelers could have infected others on board, who in turn, carried these infections to their final destinations upon reaching the US through the entry-points. Air travel within the US had also picked up during summer 2021, at least for fully vaccinated travelers (see Fig. 6f). Up until July 2021, it was largely believed that vaccines prevented the incidence of COVID-19 infections. The studies^21,22^ provided a turning point; it demonstrated that breakthrough infection rates with the Delta variant were not as low as was previously anticipated. These factors may have contributed to lack of precautionary measures adopted among passengers in US airports who came in contact with travelers from India, spreading the infection even further. Some states suffered more than the others (see Fig. 6d). During this wave, vaccination efforts were already in full swing across the US. Indeed, the rise in infections is anti-correlated with the vaccination rates in the various states (see Fig. 6e).

To back our hypothesis about migration from India being the root cause for the third wave in the US, we compare the infection growth across countries with various levels of Indian diaspora. One expects a country with a higher Indian diaspora and lower international travel restrictions to exhibit the impacts of Delta variant-based COVID-19 infections than those with a lower Indian diaspora and stricter rules on international travel. This is only true towards the beginning of the onset of Delta variant-induced infections. Indeed after the initial waves, the Delta variant reached multiple countries, irrespective of their levels of Indian diaspora. Using data from the Government of India’s Ministry of External Affairs^23^ of Indian people living overseas, we create two lists of countries, $\mathscr {H}$ with high Indian diaspora and $\mathscr {L}$ with low Indian diaspora, as follows. For all countries with a total population north of 10 million people, we compute the ratio of the Indian diaspora to the country’s population, namely *diaspora ratio*. Then, we split the countries according to the quantiles 10%, 20%, $\ldots$, 90% of the diaspora ratio. List $\mathscr {H}$ contains all countries in that list above the 90% quantile (8 countries; 4888.754 Indian people per 100,000 nationals on average). List $\mathscr {L}$ contains all the countries at or bellow the 10% quantile (9 countries; 0.979 Indian people per 100,000 nationals on average). Specifically, we choose $\mathscr {H}= \{$Australia, Canada, Malaysia, Myanmar, Nepal, Netherlands, South Africa, Sri Lanka, UK, US$\}$ and $\mathscr {L}= \{$Argentina, Brazil, China, Czech Republic, Iran, Japan, Mexico, Poland, Romania, Turkey$\}$. Our lists are as follows. $\mathscr {H}= \{$Australia, Canada, UK, Sri Lanka, Myanmar, Malaysia, Nepal, South Africa$\}$ and $\mathscr {L}= \{$Bolivia, Guatemala, Morocco, Niger, Pakistan, Somalia, Syria, Chad, Venezuela$\}$. In Fig. 7a, we plot the trajectory of the ratio of new COVID-19 cases to the country’s population for the countries in $\mathscr {H}$. In Fig. 7b, we plot the same for the countries in $\mathscr {L}$. We obtain the infection data for various countries from^24^. Note that the countries in $\mathscr {H}$ exhibit a surge of COVID-19 cases after the peak in India’s second wave on 07/05/2021. For countries in $\mathscr {H}$, the new cases median per 100,000 people before the peak (03/01/2021–05/07/2021) is 1.73 and after the peak (05/07/2021–08/15/2021) is 9.6. Likewise, the population weighted average before and after the peak are 4.47 and 14.5 respectively. On the other hand, the countries in $\mathscr {L}$ experience a decline of cases after India’s second wave peak. For the countries in $\mathscr {L}$, the new cases median per 100,000 people before the peak is 1.11, and after the peak is 1.07. In addition, the population weighted average before and after the peak are 1.88 and 2.41 respectively, an increase that is negligible. The difference in the variation of the median new cases illustrate that countries with higher Indian diaspora indeed suffered more from the initial rise of the Delta variant. For example, the UK (3,173 Indian people per 100,000 nationals), South Africa (2731 Indian people per 100,000 nationals), and the Myanmar (370,965 Indian people per 100,000 nationals), all countries with high Indian diaspora, have a surge after India’s second wave peak. On the contrary, countries with low Indian diaspora, such as Venezuela (0.46 Indian people per 100,000 nationals), Pakistan (close to 0 Indian people per 100,000 nationals), and Niger (1.24 Indian people per 100,000 nationals) exhibit a decline in the number of new cases, immediately after India’s second wave peak. Thus, the data backs the hypothesis that countries with high Indian diaspora experience initial infection growth after India’s second wave.

Instead of using a diaspora ratio at or above the 90% quantile to define $\mathscr {H}$, if we use the 75% quantile, the difference in infection rates before and after the Indian infection peak becomes insignificant. Moving downward on the diaspora ratio to define the cutoff for inclusion in $\mathscr {H}$, the difference in infection rates before and after the Indian peak becomes negligible. Reducing the cutoff on diaspora ratio for inclusion in $\mathscr {H}$ thus makes the diaspora effect disappear. Thus, the effect of Indian diaspora diminishes quickly with reduction in the diaspora ratio. This is not surprising, given that a sudden shock to the COVID-19 positivity rate in a country can only be driven by travel from India when that travel rate is high enough, which in turn, is only expected when the Indian diaspora ratio is sufficiently high. We posit that the countries in the 90% upper quantile and above experienced significant shock from the spread of the Delta variant. However, the diaspora in countries below that quantile was not high enough to witness a similar effect from the influx of the Delta variant due to travels from India.

The variation of infection *within* countries in $\mathscr {H}$ is not fully explained by the level of Indian diaspora alone. Rather, the extent of control on international travel provides a good explanation for said variation. Different countries implemented different rules to monitor and restrict international travel. For the countries in $\mathscr {H}$, we portray the level of COVID-19-related international travel control, per the database in^25^. Specifically, the international travel controls are classified between 0 and 4; 0 indicates no restrictions, 1 implies that they screen arrivals, 2 means that they quarantine arrivals from some or all regions, 3 refers to a travel ban being implemented for passengers from some regions, and 4 equates to a total border closure. Figure SM1 in Supplementary Material companion indicates that Australia, Canada and Myanmar implemented the strongest travel restrictions among the countries in $\mathscr {H}$. Interestingly, these are *exactly* the countries with the lowest levels of new infections, immediately following India’s second wave. Said differently, a tight control on international travels have proven successful to partially curb infection growth, even among countries that were more at risk to suffer from the Delta variant.

Facing a possible surge in infections from the influx of variants from other countries, a nation can adaptively choose to decide *when* to impose border restrictions. One can use a metric such as the number of districts or counties with $\ge 5$ cumulative number of COVID-19 infections from a particular date onward as in Fig. 1b to indicate a sharp rise in simultaneous infection across multiple districts/counties. Upon facing such a rise, tighter control measures for international travel are warranted to throttle further growth. Plus, restrictions on internal mobility may be necessary to arrest the growth that is already underway.

## Policy design to tackle simultaneous infection waves

This section is dedicated to outlining policy recommendations that naturally follow our data analysis in the previous sections. The recommendations follow a chronological order. We advocate various measures along the timeline of a simultaneous wave. We begin by presenting mechanisms that can quickly identify a variant of concern and prevent it from causing a national healthcare crisis.

We emphasize that all our policy recommendations are based on analysis performed on publicly available data, the quality over which we have no control. There can be systematic biases in the data that vary with location and time. See^26,27^ for examples of the effect of reporting delays and possible ways (via a technique called nowcasting) to mitigate its impacts on subsequent data analysis. Also, see^28^ for alleged under-reporting of COVID-19 cases in various countries. While we do not directly control for such effects and relegate more careful analysis to future work, our associative studies illustrate the need to accommodate the different wave patterns of epidemic spreads and make appropriate policy decisions.

### Perform genomic testing

Figure 1a illustrates that COVID-19 infections began rising in Maharashtra in early 2021–a trend that was not apparent in other states. Such a growth, which is not corroborated by data from other states, could have served as a warning sign, especially when juxtaposed with the information that a random mutation (B.1.617) of the virus was discovered there in late 2020. Akin to other viruses, SARS-CoV-2 will likely continue to mutate. Whenever a region shows heightened growth in infections compared to neighboring regions, one must carefully consider the possibility of a new mutation. Detection of said mutation relies on adequate testing (both symptomatic and asymptomatic), followed by genomic sequencing, as suggested by^9^. India had only identified 13,000 genetic sequences (0.5/1000 cases) compared to the United States (US), which had identified 400,000 sequences (12/1000 cases) around 05/05/2021^29^. Considering that sequencing rates in the US had been deemed sub-optimal (see^30^), India’s sequencing rate appears significantly low; see^8,31^ for details. Because different nations have varied abilities to test and sequence, a global initiative must be undertaken to quickly detect such variants and proactively engage in risk mitigation strategies. Otherwise unchecked, such variants are bound to percolate to other regions. The Delta variant indeed became the driver of new COVID-19 infections worldwide in 2021, delaying the efforts to reopen the economies across the globe. Limited sequencing capabilities, albeit increasing fast, in various countries continue to pose challenges in effectively assessing the risks of circulating mutant variants.

**Figure 8:**
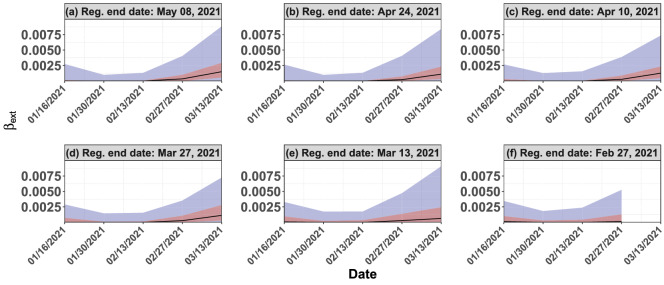
Plots (**a**)–(**f**) capture the quantiles of $\beta _ext$’s across Indian districts over two-week time windows from 01/16/2021 to 03/13/2021. Regression of Plots (**a**)–(**f**) uses data until 05/08/2021, 04/24/2021, 04/10/2021, 03/27/2021, 03/13/2021, and 02/27/2021, respectively.

### Avoid or manage super-spreaders

Our data analysis suggests that the baths at Kumbh Mela was the likely super-spreader event that was responsible for spreading the Delta variant across India. The obvious step to mitigate infection spread during a pandemic is to stop such potential super-spreader events proactively. Especially in a country with low vaccination rates. India has vaccinated $\sim$47% of the population with at least one dose by 10/01/2021. The same rates prior to the Kumbh Mela festival, however, were much lower at $\sim$4.7%. Super-spreader events can lead to a national healthcare crisis when combined with the rise of a new variant. A particularly successful example of an international gathering that did not have such dire consequences is the 2021 Tokyo Olympics. Japan witnessed a sharp rise in infection growth following the Olympics. However, its impact as an international super-spreader event appears limited. The key difference between the Olympics and Kumbh Mela is the rate, accuracy, and auditing of testing protocols. While Olympic staff and athletes were required to adhere to a stringent testing regimen, India’s problems were compounded by possibly fake coronavirus tests^32^ that were administered prior to the baths at the Kumbh.

### Detect a simultaneous wave early

Once a super-spreader event happens, its impact on infection load becomes unavoidable and imminent. However, an early warning system to detect such a rise in infections can prove helpful to plan forward in order to tackle the healthcare challenges. We now explore the development of such an early warning system using our estimation procedure on India’s second wave data. Recall that we estimated $\beta _\text {int}$ and $\beta _\text {ext}$ for multiple regions by minimizing $\varphi$ defined in (5). Note that in Fig. 8, $\beta _\text {ext}$ grows rapidly around April 21, despite the explicit penalty on $\beta _\text {ext}$’s in the third summand of $\varphi$. In other words, a sharp rise in $\beta _\text {ext}$ serves as an indication that the epidemiological diffusion model does not explain the data; rather, it is a signature of a super-spreader event. How much evolution of a wave is necessary to discover such a signature rise in $\beta _\text {ext}$? To that end, we minimize $\varphi$ using data up to various end-dates and compare the quantiles of $\beta _\text {ext}$’s computed over the time window from 01/16/2021 to 03/13/2021. We choose the progressively earlier end-dates of 05/08/2021, 04/24/2021, 04/10/2021, 03/27/2021, 03/13/2021, and 02/27/2021 and plot said quantiles in Fig. 8a–f. The quantiles over the defined time-window are similar in all plots, revealing that the parameter estimation procedure is quite robust to the choice of end-dates. For end-dates later than 03/13/2021, the quantiles of $\beta _\text {ext}$ evaluated on that date rise sharply compared to the quantities earlier in the time-window. In other words, the wave could have been detected as early as 03/13/2021 using our estimation procedure (not so much on 02/27/2021, however). Given India’s healthcare challenges during April and May 2021, an early warning on March 13 could have allowed policymakers to prepare better and potentially avoid resource shortages.

### Monitor international travel at entry ports

India’s second wave started to slow down in the second half of May, thanks to the significant decrease in mobility caused by the partial to total lock-downs. Most Indian states locked down in early May, while some, including Delhi and Maharashtra, implemented it earlier in April. Lock-downs have substantial economic impacts, and typically, they adversely affect vulnerable socio-economic classes. However, temporary targeted and planned lock-downs may be the only viable solution available to arrest the rapid growth of the infection, given the rising death toll and the shortage of medical supplies to care for the infected. Moreover, a simultaneous rise in infections across the nation, measured by the speed of the growing number of districts with $\ge 5$ infections (as in Fig. 1b), can serve as a signal to impose early restrictions on mobility. Left unchecked, a meteoric rise in infections across a nation puts enormous burdens on the national healthcare system. Early lock-downs, planned using scenario models, can prevent such a rise and avoid severe resource shortages.

Similar to the role of lock-downs in intra-country infection growth, international travel controls are vital to mitigate the circulation of problematic mutations across a nation, especially from those countries with a rising level of variant-induced infections. Our analysis showed that limiting the influx of infections from international travel had successfully avoided a large wave in countries such as Australia, Myanmar, and Canada. Per our analysis, countries with high levels of Indian diaspora were indeed at higher risks for infection due to the Delta variant. However, stringent international border closures helped the above three countries to circumvent a Delta wave. We fully recognize that international travel closures have socio-political connotations and impose heavy economic burdens. Rather than imposing blanket travel bans, a more targeted approach such as testing every arriving passenger at entry ports and imposing a temporary isolation before test results arrive, can prove useful. Such a careful approach towards international travel is paramount to combat an epidemic in an increasingly interconnected world.

### Vaccinate in bulk

As of May 24, 2021, 11.2% of India’s population had received at least one dose of the vaccine, and 3.1% were been fully vaccinated, using a combination of the University of Oxford-AstraZeneca vaccine (manufactured as Covishield by the Serum Institute of India) and Covaxin (manufactured by Bharat Biotech). Early warning signs of an abnormal infection growth in one area can be used to perform targeted vaccinations to prevent the rise of and suffering from a transmissible and/or virulent variant. Given the timeline of vaccine production and dissemination in India and elsewhere, it is unlikely that vaccination could have been used preemptively to mitigate India’s second wave. However, vaccination has emerged as a very effective tool to combat COVID-19. Figure 6e shows that vaccination rate was anti-correlated with COVID19 infection rates across the states in the US on 08/15/2021. Given the effectiveness of the vaccines in at least curbing the severity of infections from the rising variants (including Omicron), we believe that vaccination remains the *only* long-term solution to counter this pandemic.

## Conclusions

Some waves of COVID-19 infections have grown simultaneously across a nation, overwhelming its national healthcare system. India’s second wave of COVID-19 in 2021 is a prime example of such a simultaneous wave across the country. Our data analysis from 394 districts in India suggested that a super-spreader event had amplified the rise of the highly transmissible Delta variant. Kumbh Mela was argued as the likely candidate event that caused the spread.

A multi-area SIR epidemic model was developed whose estimation process was shown to be able to pick up the shock due to a super-spreader, shortly after the event had happened. Therefore, this estimation procedure has the potential to serve as an indicator of a future imminent simultaneous wave. While we believe our techniques and conclusions will hold generally to study simultaneous waves, our results are primarily derived using the infection data from India. Such observational data has limitations and the conclusions need further validation.

Oddly enough, data from the third wave in the US in the summer of 2021 also exhibited simultaneous rise across the different states. Our data analysis supported the hypothesis that international travels from India might have been the root cause behind this rise. Specifically, countries with higher Indian diaspora showed surges in COVID-19 infections, immediately following India’s peak–a trend opposite to that in countries with low Indian diaspora. Strict border protocols appear to have mitigated such surges. Absent such controls or close monitoring, a simultaneous wave due to a super-spreader in one country can seed simultaneous waves in other countries.

Based on our analysis, we suggested four specific policy recommendations during the whole lifetime of a simultaneous wave. First, one must check for the anomalous rise of infections in one region to quickly identify potent variants of concern. Second, any large congregation must either be avoided or strict testing protocols should be followed in such events to prevent a said event from becoming a super-spreader. Third, one must look for early signs of a simultaneous wave, following any large congregation, through statistical tests to prepare early for an imminent impact. Fourth, international travel should be carefully monitored during the rise of a variant in any part of the world. While these recommendations are specific to combat simultaneous waves of infections, vaccination in bulk holds the key to restore pre-pandemic life. These recommendations are timely, given the rise of newer variants such as the Omicron and the IHU.

### Ethics approval

This approval does not apply as we did not work with patient data.

## Supplementary Information

Supplementary Information.

## Data Availability

All datasets and the code for the analysis are available in https://github.com/heart-analytics/COVID19-India.
