# Supplementary material for: Detecting and mitigating simultaneous waves of COVID-19 infections
Source: Sci Rep. 2022 Oct 6;12:16727. doi: 10.1038/s41598-022-20224-5 (PMC9537162; doi:10.1038/s41598-022-20224-5)
Supplement: Supplementary file 1 — Supplementary Information. [file 41598_2022_20224_MOESM1_ESM.pdf]

Supplementary Material of manuscript *Detecting and Mitigating Simultaneous Waves of COVID-19 Infections*

S. Souyris, S. Hao, S. Bose, A. C. III England, A. Ivanov, U. K. Mukherjee, and S. Seshadri

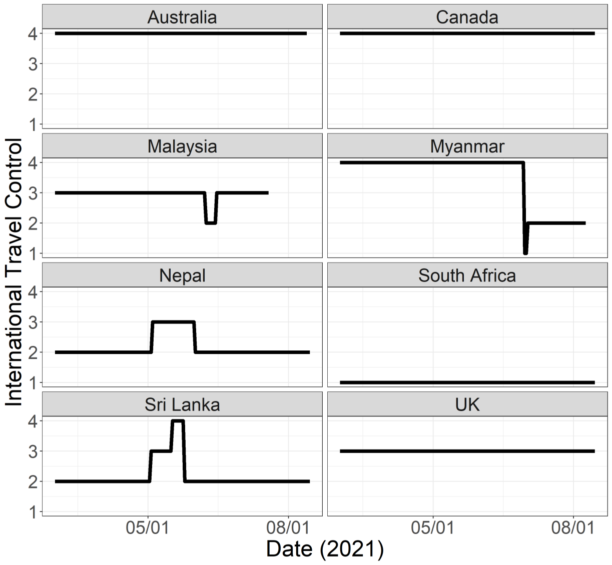

Figure SM1. Travel restrictions for countries with high Indian diaspora, $\mathscr{H}$ (0=no restrictions, 1=arrival screening, 2=quarantining arrivals from some regions, 3=partial travel ban, and 4=total border closure).
